# Using Design Thinking Principles to Improve Outpatients’ Experiences in Hospital Pharmacies: A Case Study of Two Hospitals in Asir Region, Saudi Arabia

**DOI:** 10.3390/healthcare9070854

**Published:** 2021-07-06

**Authors:** Dalia Almaghaslah, Abdulrhman Alsayari, Saleh Ali Alyahya, Rana Alshehri, Khawlah Alqadi, Sumiah Alasmari

**Affiliations:** 1 Department of Clinical Pharmacy, College of Pharmacy, King Khalid University, Abha 61441, Saudi Arabia; 2 Department of Pharmacognosy, College of Pharmacy, King Khalid University, Abha 61441, Saudi Arabia; alsayari@kku.edu.sa; 3 Medical Supportive Services, Health Affairs, Asir Region, Ministry of Health, Abha 61441, Saudi Arabia; saaalyahya@moh.gov.sa; 4 College of Pharmacy, King Khalid University, Abha 61441, Saudi Arabia; rainshehri@gmail.com (R.A.); Khawlah1996@gmail.com (K.A.); sumia203@hotmail.com (S.A.)

**Keywords:** design thinking, hospital pharmacy, Saudi Arabia

## Abstract

*Introduction:* Design thinking, an innovative problem-solving approach, has gained wide popularity in healthcare disciplines. The aim of this work is to improve outpatients’ experiences in hospital pharmacies in two hospitals in Asir region, Saudi Arabia. *Methods*: The design thinking approach, adopted from Stanford University’s D-School, was used in this study. *Results*: Several problems were identified: lack of comfortable environment in the pharmacies’ waiting area, lack of a queue management system, and workflow inefficiencies related to ordering and supplies of medicines. A prototype was proposed to overcome these challenges. *Discussion and Conclusion*: The design thinking approach helped in identifying end-user (patients visiting outpatient pharmacies) values and desires and provided an understanding of their struggles. It also proposed tailored solutions that could improve patients’ experiences while using the services of the outpatient pharmacies.

## 1. Introduction

Design thinking is defined as a systematic approach that prioritizes deep empathy for users’ desires, needs, and challenges to fully understand a problem, with the aim of developing more comprehensive and effective solutions [1,2] Others define design thinking as a solution-based approach using an iterative process that seeks to understand the user, challenge assumptions, and envision problems differently. The goal of this process is to identify alternative strategies and solutions that are not apparent initially.

Design thinking includes the following stages: empathy, define, ideation, prototype, and testing [1,3].

Empathy is the first and most important step, where a deep, diverse understanding of the user group’s needs, values, and desires is established. It may also include observation, which requires engaging with the user group by observing the behaviors that occur in their environment with self-documentation, such as taking pictures or recording audios or videos. Define or interruption includes describing the point of view of and needs of user group. Ideation) involves generating solutions by combining empathy and observation; this usually requires a multidisciplinary team to address a complex issue. The prototype phase involves generating and testing multiple alternative solutions before finally selecting the best solution through an iterative process. testing involves obtaining feedback about the prototypes from the user group, transferring empathy, as well as refining the solutions [1,4,5].

Design thinking has been used by various disciplines, including healthcare [6]. The literature showed many examples of successful use of the design thinking framework for problem solving. For example, it was used to improve patient experiences [6,7]; address the challenges heath care providers faced in a nursing home [2]; re-design a hospital pharmacy management system [8]; enhance psychological interventions [9]; improve therapeutic outcomes, medication information, and self-management for diabetic patients [10,11]; and in health education [12].

An evaluation of hospital pharmacy practices is essential to assure that they meet the global standards and to prioritize practice advancements [13]. An evaluation of hospital pharmacy practice was first conducted in 1998 by the American Society of Health-System Pharmacists (ASHP) using a national survey [14,15]. The survey examined the role of the pharmacist in distinct stages: prescribing, transcribing, dispensing, administrating, monitoring, and educating patients. In 2005, the International Pharmaceutical Federation (FIP) also highlighted the importance of evaluating hospital pharmacy services by conducting a multinational survey involving 192 countries. Most recently, in 2010, the European Association of Hospital Pharmacists (EAHP) implemented a survey concerned with the same area of practice [14,16,17].

In Saudi Arabia, the hospital pharmacy is the second largest pharmacy sector, employing 18% of the total pharmacy workforce [18]. The country’s pharmacy sector is considered relatively advanced compared to neighboring countries. The role of the pharmacist in hospital settings includes medication verification, dispensing, management of medication supply and storage, drug information services, as well as supervision of pharmacy interns. Other more specialized practice areas include chemotherapy, parenteral nutrition, and sterile medication preparation. Pharmacists are also responsible for reporting adverse drug events (pharmacovigilance). The performance indicators used to evaluate hospital pharmacy services include patient satisfaction, patient waiting time, number of prescriptions filled, and number of dispensing errors [19].

Hospital pharmacy services in the capital city, Riyadh, were assessed previously by Alsultan and colleagues [14,16,17], while those in Jeddah were evaluated by Altayar and colleagues [15]. However, the previous studies evaluating hospital pharmacy services in Saudi Arabia utilized objective tools, surveys that did not take into consideration end-users’ needs, desires, and values. They were also conducted in the largest and most developed cities in the country, so their findings might not be generalizable to other regions. Hence, the current study was conducted. The aim of this study was to evaluate patient experiences while visiting outpatient pharmacies in two hospitals in the Asir region of Saudi Arabia using design thinking approaches. This would lead to the development and testing of strategies to overcome all identified challenges.

## 2. Methodology

An empirical investigation was conducted to understand the context of hospital pharmacy services using the design thinking approach, adopted from Stanford University’s D-School. A case study from two hospitals in Asir Region, Saudi Arabia was used in this study to identify the most important issues.

**Figure 1:**
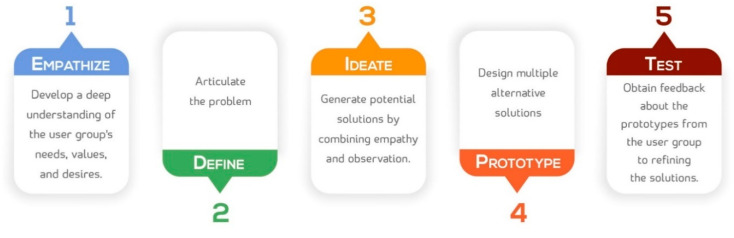
Stages for developing a design pattern.

The study was conducted between January and March 2020, semi-structured interviews (preset open-ended questions, Table 1) were conducted with outpatients utilizing the outpatient pharmacy services in two hospitals in Asir. The interviews were conducted by a female researcher who had previously worked on qualitative studies examining health services in the country. Participants were recruited through a convenience sample. A total of 41 patients agreed to participate in the study (Table 2). Written informed consent was obtained from the participants before the interviews were conducted. Participants were also informed that their identities would remain anonymous. Detailed topic guides were developed and piloted with other patients prior to the interviews. The interviews ranged from 15 to 20 min in length and were conducted in Arabic. The interviews were tape recorded, transcribed verbatim shortly after being completed, and then translated into English. To ensure the quality of the translation, four of the interview scripts were double checked by a second English-Arabic bilingual investigator at the King Khalid University (KKU), College of Pharmacy. Ethical approval for this study was granted by the Ethical Committee of Scientific Research at KKU (ECM 2020-221; HAPO-06-B-001). The methodology is structured in five main stages, as illustrated in Figure 1.

### 2.1. Stage I (Empathize)

At the beginning of the case studies, the researcher interacted with patients visiting the outpatient pharmacies in the two hospitals in Asir. Based on the participants’ perspectives and pain points, a list of key problems was identified through the following steps.

**Observation:** Researchers shadowed the patients and observed their behavior during their visits to the outpatient pharmacy. Observations of patients in real-life contexts, along with the interviews, provided insights into the struggles patients encounter while utilizing outpatient pharmacy services.

**Engaging:** Several one-to-one semi-structured interviews were conducted in the pharmacies’ waiting areas. To deeply engage with patients, it was essential to allow them to lead the conversation and to elaborate on their points in order to fulfill the exploration nature of the design thinking approach.

**Watching and Listening:** Observation and engagement were combined, for instance, by asking patients of their experiences with the waiting time and dispensing of their medications. Pictures of the hospital pharmacies and waiting areas were also taken. Through several storytelling activities, the key issues with the pharmaceutical services process and flow raised questions on ways to improve their experience.

### 2.2. Stage II (Define)

After collecting information regarding the core issues experienced by patients, the final key issues were defined using the two following steps.

**Contextualize:** The key themes that emerged from the interviews, as well as those related to the research questions asked during the empathize stage, were combined into a set of draft analytic frameworks. Drafts were tested with a small amount of interview data and the final themes were used to code the data. Two investigators coded the data and a total of four themes (problems) were identified and are listed below.

**Synthesize:** During this phase, the possible solutions were identified. This was completed by synthesizing and prioritizing the core needs. The end-user (patient) perspective was structured by combining three key components—user, need, and insight—into an actionable problem statement.

### 2.3. Stage III (Ideate)

The previous stage (define) directed the researchers’ efforts to generating possible solutions to overcome the core problems identified through the following steps.

**Create:** Stakeholders’ expertise (pharmacy, information technology, and interior design) was combined through a series of brainstorming sessions. This allowed the synergy of the stakeholders to reach promising solutions. Adding limitations, including inspiring resources and accepting misunderstanding facilitated the development of creative ideas on improving patient experiences in outpatient pharmacies.

**Prototype:** Prototypes were formed to support the ideation phase. Several development stages enabled the creation of new ideas. This stage used a few ideation techniques, including brainwriting, provocation, storyboard, and mind mapping.

**Separate**: Evaluation of ideas was discouraged to allow the generation of more inspiration and creativity.

### 2.4. Stage IV (Prototype)

The collected information directed the possible solutions and strategies. The core issues and required solutions were determined, then various prototypes were developed through the following steps.

**Build:** The model process was started, i.e., constructing something or coding basic solutions, as a useful start towards a prototype.

**Variables:** Each prototype was designed to tackle a certain problem when evaluated as to the ways it enhances the patient experience in the pharmacy.

**Build from insights gained**: The prototypes were clearly identified and the patient behavior towards the changes will be monitored. Feedback to be collected in the testing phase.

### 2.5. Stage V: Testing (Validation with Collaborators)

The last stage of design thinking involves testing the proposed solutions that address the core issues and needs.

**Observe and Refine:** All solutions to be implemented for the end-user (patients) will be observed and patient feedback received.

**Create Experiences**: The newly implemented features will be tested by noting the way that patients experience them, rather than by their evaluation

## 3. Result

### 3.1. Stage I (Empathize) and Stage II (Define)

**Figure 2:**
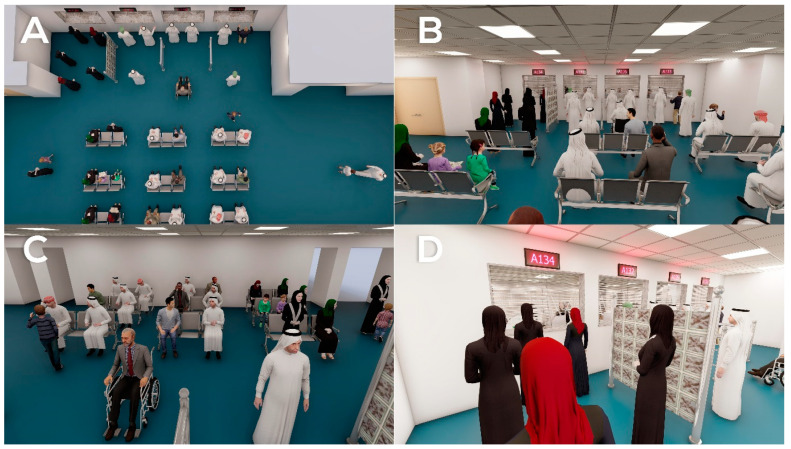
Pharmacy waiting area in hospital 1. (**A**–**C**): An overview of the pharmacy waiting area from different angles, showing a lack of comfortable environment. (**D**): Number of windows serving male patients in comparison with female patients, indicating a lack of equity in waiting times between the two genders.

**Figure 3:**
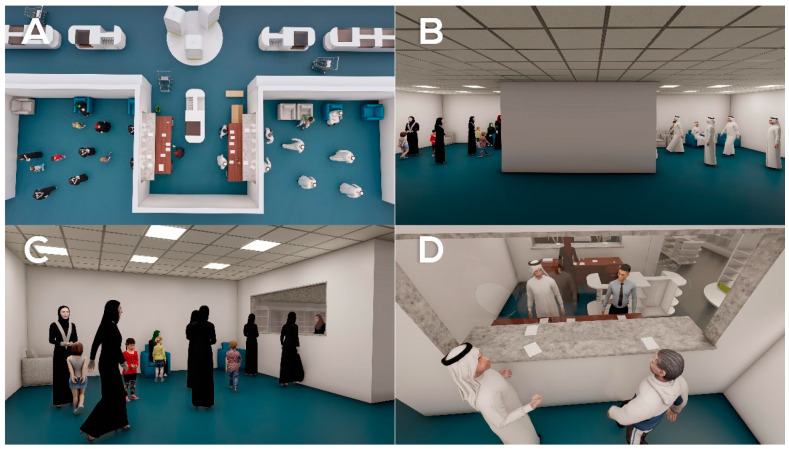
Pharmacy waiting area in hospital 2. (**A**,**B**): An overview of the pharmacy waiting area from different angles, showing lack of comfortable environment in the pharmacy’s waiting area. (**C**): Lack of queue management system in the hospital, causing improper queue management and overcrowded waiting area. (**D**): Use of hand-written prescriptions, indicating workflow inefficiencies through ordering and supply of medicines that is slow to arrive between physicians and pharmacies (lack of e-prescribing).

Based on the participants’ perspectives and pain points, a list of key problems (Table 3) were identified as follows:

1. Lack of comfortable environment in the pharmacies’ waiting areas (limited seating, lack of refreshments, poor lighting, lack of adjustment for disabled patients) in both hospitals 1 and 2 (Figure 2 and Figure 3).
2. Lack of a queue management system in hospital 2, causing improper queue management (Figure 3C).
3. Lack of equity in waiting times between the two genders (more windows serving male patients than females) in hospital 1 (Figure 2C).
4. Workflow inefficiencies through ordering and supplies; medicines are slow to arrive between physicians and pharmacies, and patients are using hand-written prescriptions in hospital 2 (lack of e-prescriptions) (Figure 3D).

### 3.2. Stage III (IDEATE) and Stage IV (Prototype)

The design thinking approach helped to identify and prioritize patients’ needs related to software and interior design solutions, which can be summarized as follows.

**Figure 4:**
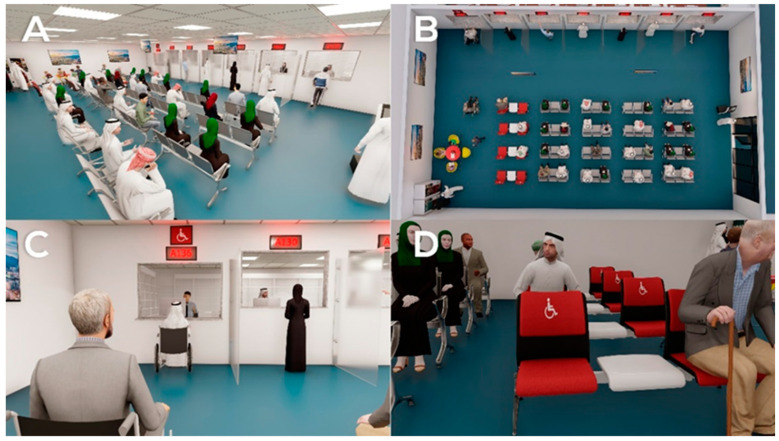
The final prototype I. (**A**,**B**): An overview of the pharmacy waiting area from different angles. (**C**,**D**): Arrangements for disabled patients.

**Figure 5:**
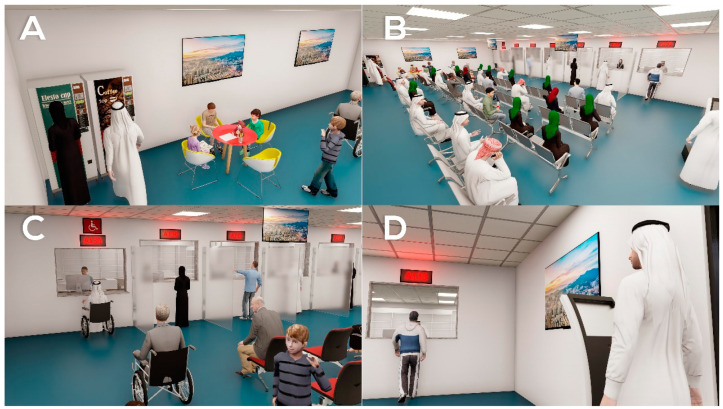
The final prototype II. (**A**): Children’s play area and vending machines. (**B**): TV screen. (**C**): Partitions separating windows. (**D**): Queue management system.

- **Software solutions:**
  ○ Introduce electronic-prescribing initiatives for physicians so they can communicate directly with the pharmacy in hospital 2
  ○ Establish a queue management system in hospital 2.
- **Interior design solutions:**
  ○ Redesign the pharmacy waiting areas, increasing the number of seats allotted for female patients in both hospitals 1 and 2 (Figure 4A,B).
  ○ Introduce adaptive arrangements for disabled patients (i.e., wheelchair accessible space in waiting rooms, dispensing windows to be height appropriate for wheelchair users in both hospitals (Figure 4C,D).
  ○ Provide vending machines for refreshments in both hospitals (Figure 5A).
  ○ Establish children’s corner in the waiting room in in both hospitals (Figure 5A).
  ○ Provide TV **screens** in **waiting rooms** in both hospitals (Figure 5B).
  ○ Brighten the dark waiting area in hospital 2 (Figure 4A,B).
  ○ Provide partitions at the end of the counter (Figure 5C).

### 3.3. Stage V: Testing (Validation with Collaborators)

Focusing on the two main outcomes, the design thinking approach was integrated. Software solutions, as well as interior design solutions, were tested to address the identified healthcare needs. The aim was to overcome the shortcomings in both the software system and the interior design of the pharmacies. Having identified the major issues experienced by the patients, the possible solutions were explored further. The proposed solution to the interior design shortcoming addresses the patients’ needs regarding seating capacity, waiting times, and arrangements for disabled patients. The proposed solution to the software issues addresses the patients’ needs for a queue management system and a system that monitors and tracks drug-flow within the hospital system. The final single prototype was a model design that overcomes all the shortcomings in both hospitals, as shown in Figure 4

## 4. Discussion

Design thinking is a framework used to solve end-user problems [2]. This approach provides in-depth understanding of the user’s feelings, challenges and values. This innovative technique was incorporated here to overcome the shortcomings experienced by patients with the pharmaceutical services provided in two hospitals. Hence, it provided an opportunity for stakeholders to further improve the patients’ experiences within the pharmacy (Carroll, 2016).

In this case study, pharmaceutical services were explored in two tertiary hospitals in the Asir region of Saudi Arabia. This paper describes the need to facilitate deep understanding of patients’ “realities” in the context of the services they receive during their visits to the outpatient pharmacies, thus identifying the need for software and interior design solutions.

Previous studies evaluating hospital pharmacy services used more objective quantitative evaluation tools that do not consider end users’ needs. Hence, these problem-solving frameworks might have not reached the same conclusions. For example, an understanding of cultural influences in interior design, atmosphere, and patient satisfaction is crucial. In this context, cultural barriers require certain layouts that separate the waiting areas for each gender. This also applies to the filling of prescriptions; females prefer to get their prescriptions dispensed from the “female only” window that is served by female pharmacists. Initially, there was only a single female service window compared to three male service windows, so the waiting time for females was longer.

Another issue was the lack of electronic systems in one of the two hospitals. This affected patients’ satisfaction as it caused disorganization in filling prescriptions, long wait times, and more difficult communication between pharmacists and prescribers. For example, if a prescribing error is identified by the pharmacist, the inquiry cannot be sent directly to the physician. Hence, the patient is required to go back the physician, which was challenging for some patients, especially the elderly.

Special attention was directed to the needs of children by providing a play area for them, while the elderly were allotted seating priority. Disabled patients were provided with spaces in the seating area designated for wheelchair users, as well as with improved physical accessibility to the service window. Other general improvements to the waiting areas were achieved by brightening the room and providing vending machines, TV screens and a queue management system.

This work highlighted the gap between healthcare stakeholders, software engineering stakeholders, and the interior design community when identifying and addressing healthcare issues. The design thinking approach narrowed the gap by bringing together the perspectives of a multidisciplinary team. This research showed that patients are generally satisfied with their interactions with pharmacists, but their overall experience could be upgraded by improving the delivery of services (workflow) and the surrounding environment. Hence, the expertise of a healthcare team, software engineers, and interior designers contributed to the design thinking framework, facilitating an innovative design.

One limitation of this research was its narrow scope of only two single case studies, with each outpatient pharmacy having a different list of problems. Therefore, the findings might not be generalizable to other settings. The proposed prototypes were implemented only in virtual forms (computer-aided design), with a testing phase to be conducted in the future.

## 5. Conclusions

This research shows that design thinking can provide innovative solutions, technology-enabled pharmaceutical services delivery and interior design solutions to overcome issues affecting the patient experience within outpatient pharmacies.

## Figures and Tables

**Table 1:** Interview guide.

No.	Questions
1	How was your experience in the outpatient pharmacy today? Could you tell us more?
2	How were the services? Waiting area, waiting time etc.?
3	Can you elaborate?
4	How was the pharmacist?
5	Was there any particular thing that you liked/disliked?
6	How would your experience be better?
7	Out of ten, how would you rate the service today?

**Table 2:** Hospitals and participants information.

Hospitals and Participants Information	Hospital 1	Hospital 2
Hospital type	Government	Government
Level of expertise	General tertiary	Specialized
Number of beds	600	300
Number of interviews	21	20
Gender of participants	Male = 8 Female = 13	Male = 8 Female = 12
Age of participants		
20–29	4	2
30–39	6	8
40–49	3	4
50–59	4	3
>60	4	3
Reason for visiting		
Patient	18	6
Caregiver	3	13
Visitor	-	1

**Table 3:** Health Innovation framework.

End-User Prospective	Key Problem	Hospital	Key Management
**Patient 10 (M)** I suggest expanding the female waiting area as sometimes there are no seats inside the female designated area, so I have sat in the common seats with men around. **Patient 4 (M)** I wish there was a number waiting system **Patient 15(A)** The waiting area is overcrowded and full with patients sometimes.	Lack of queue management system, causing improper queue management and overcrowded waiting area.	Hospital 2	Waiting system to be implemented. The waiting area to be expanded.
**Patient 14 (A)** There is not enough space in the pharmacy waiting area, and there is only a single female dispensing windows. **Patient 2 (A)** There is only one window for females compared to three for males which makes the place packed with people.	Female patients experience of longer waiting times (lack of equity in waiting times between the two genders).	Hospital 1	Dispensing prescriptions for both male and female patients can be done from any window regardless of the gender.
**Patient 2 (M)** There are not enough seats and the waiting area is gloomy and depressing. **Patient 15(A)** The waiting area is very small and cannot accommodate the large number of patients There are no refreshments or even drinking water. I would suggest adding TV screens and kids play corner to entertain patients while waiting for their prescriptions. **Patient 16(A)** There is no drinking water for me to take my medications while I’m here. **Patient 4 (M)** The chairs are wobbly and not in suitable condition, the room is dark. **Patient 3 (M)** It would be great to have free refreshments like tea or coffee. **Patient 12 (M)** There is not enough space for my son’s wheelchair.	Lack of comfortable environment in the pharmacies waiting area (limited seating, lack of refreshments, poor lighting, lack of adjustment for disabled patients)	Hospital 1, 2	The pharmacy waiting areas to be redesigned with more seating allotted for female patients. Arrangements for disabled patients (i.e., wheelchair accessible space) to be made available for wheelchair users to park while waiting for their prescription, dispensing windows to be height appropriate for wheelchair users. Vending machines to be installed. Dim lighting be replaced with brighter lighting. TV screens to be implemented. Children’s play corner to be established.
**Patient 15 (M)** I wish there was a direct communication between the physician and the pharmacist as well as number waiting system. **Patient 4 (M)** It would be great if electronic prescribing was implemented as it would reduce the medication errors, and order for prescription filling	Workflow inefficiencies through ordering and supply of medicines that is slow to arrive between physicians and pharmacists (lack of e-prescribing)	**Hospital 2**	A system that monitors and tracks drug-flow within a hospital system to be installed.

## Data Availability

The data presented in this study are available on request from the corresponding author.

## References

[1] Roberts J.P., Fisher T.R., Trowbridge M.J., Bent C. (2016). A design thinking framework for healthcare management and innovation. Healthcare.

[2] Eines T.F., Vatne S. (2018). Nurses and nurse assistants’ experiences with using a design thinking approach to innovation in a nursing home. J. Nurs. Manag..

[3] Chan K. (2018). A Design Thinking Mindset Beyond the Public Health Model. World Med. Heal. Policy.

[4] Valentine L., Kroll T., Bruce F., Lim C., Mountain R. (2017). Design Thinking for Social Innovation in Health Care. Des. J..

[5] McLaughlin J.E., Wolcott M.D., Hubbard D., Umstead K., Rider T.R. (2019). A qualitative review of the design thinking framework in health professions education. BMC Med. Educ..

[6] Taisuke U., Carl K. (2009). Using design thinking to improve patient experiences in Japanese hospitals: A case study. J. Bus. Strategy.

[7] Kim S.H., Myers C.G., Allen L. (2017). Health care providers can use design thinking to improve patient experiences. Harv. Bus. Rev..

[8] Chanpuypetch W., Kritchanchai D. A design pattern for modelling and simulation in hospital pharmacy management. Proceedings of the 31st European Conference on Modelling and Simulation, ECMS 2017.

[9] Yeager D.S., Romero C., Paunesku D., Hulleman C.S., Schneider B., Hinojosa C., Lee H.Y., Brien J.O., Flint K., Roberts A. (2016). Using design thinking to improve psychological interventions: The case of the growth mindset during the transition to high school. J. Educ. Psychol..

[10] Henderson V.A., Barr K.L.C., An L.C., Guajardo C., Newhouse W., Mase R., Heisler M. (2013). Community-based participatory research and user-centered design in a diabetes medication information and decision tool. Prog. Community Heal. Partnersh. Res. Educ. Action.

[11] Yu C.H., Parsons J.A., Hall S., Newton D., Jovicic A., Lottridge D., Shah B.R., Straus S.E. (2014). User-centered design of a web-based self-management site for individuals with type 2 diabetes—Providing a sense of control and community. BMC Med. Inform. Decis. Mak..

[12] Pugliese R.S., Girone G. (2018). Design Thinking In Pharmacy Education: Inspiring Creative Problem Solving in the Next Generation of Pharmacists. Inov. Pharm..

[13] Al Sabban H., Al-Jedai A., Bajis D., Penm J. (2018). The Revised Basel Statements on the Future of Hospital Pharmacy: What Do They Mean for Saudi Arabia?. Int. J. Pharm. Pract..

[14] Alsultan M.S., Khurshid F., Al-jedai A.H., Mayet A.Y. (2012). Hospital pharmacy practice in Saudi Arabia: Dispensing and administration in the Riyadh region. Saudi Pharm. J..

[15] Altyar A.E., Sadoun S.A., Alradadi R.S., Aljohani S.S. (2019). Evaluating Pharmacy Practice in Hospital Settings in Jeddah City, Saudi Arabia: Prescribing and Transcribing—2018. Hosp. Pharm..

[16] Alsultan M.S., Khurshid F., Salamah H.J., Mayet A.Y., Al-jedai A.H. (2012). Hospital pharmacy practice in Saudi Arabia: Prescribing and transcribing in the Riyadh region. Saudi Pharm. J..

[17] Alsultan M.S., Mayet A.Y., Khurshid F., Al-jedai A.H. (2013). Hospital pharmacy practice in Saudi Arabia: Drug monitoring and patient education in the Riyadh region. Saudi Pharm. J..

[18] Almaghaslah D., Alsayari A., Asiri R., Albugami N. (2018). Pharmacy workforce in Saudi Arabia: Challenges and opportunities: A cross-sectional study. Int. J. Health Plann. Manag..

[19] Al-Jedai A., Qaisi S., Al-Meman A. (2016). Pharmacy practice and the health care system in Saudi Arabia. Can. J. Hosp. Pharm..

